# MODIFICATION OF THE ASSOCIATION BETWEEN HEARING LOSS AND DEMENTIA PREVALENCE BY SELF-REPORTED SLEEP DISTURBANCES

**DOI:** 10.1093/geroni/igad104.0630

**Published:** 2023-12-21

**Authors:** Kening Jiang, Adam Spira, Nicholas Reed, Alison Huang, Frank Lin, Jennifer Deal

**Affiliations:** Johns Hopkins Bloomberg School of Public Health, Baltimore, Maryland, United States; Johns Hopkins Bloomberg School of Public Health, Baltimore, Maryland, United States; Johns Hopkins Bloomberg School of Public Health, Baltimore, Maryland, United States; Johns Hopkins Bloomberg School of Public Health, Baltimore, Maryland, United States; Johns Hopkins, Baltimore, Maryland, United States; Johns Hopkins University, Baltimore, Maryland, United States

## Abstract

Hearing loss and sleep disturbances are both known risk factors for dementia. High prevalence and modifiability in late life make them potential targets for dementia prevention. However, their combined contribution to dementia is unknown. The National Health and Aging Trends Study (NHATS) is a longitudinal panel survey of a nationally representative sample of Medicare beneficiaries. A validated algorithm based on self- or proxy-reported diagnosis, AD8 dementia screening, and cognitive tests was used to classify participants as having probable/possible vs. no dementia. Pure-tone audiometry was added to the 2021 round of NHATS; participants with better-ear 4-frequency (0.5, 1, 2, 4 kilohertz) pure-tone average above 25 decibels were categorized as having hearing loss. Participants reported their frequencies of taking >30 minutes to fall asleep and having trouble falling back asleep and were defined as having each symptom if it was experienced ≥2 nights/week. We used Poisson regression with robust standard errors and included an interaction term between sleep disturbances and hearing loss, adjusting for demographics, smoking, body mass index and comorbidities. Among 2,383 participants aged 71+ years (46% male, 7% Black), hearing loss was cross-sectionally associated with higher dementia prevalence only among participants with sleep disturbances (>30 minutes to fall asleep: prevalence ratio [PR]=1.81, 95% CI:0.99, 3.31; difficulties falling back asleep: PR=1.82, 95% CI:1.02, 3.26). Older adults with both hearing loss and sleep disturbances might represent a high-risk subgroup that needs to be prioritized. With the complex etiology of dementia, a better understanding of the co-occurrence of multiple risk factors is needed.

